# Expanding the gene pool for soybean improvement with its wild relatives

**DOI:** 10.1007/s42994-022-00072-7

**Published:** 2022-05-20

**Authors:** Yongbin Zhuang, Xiaoming Li, Junmei Hu, Ran Xu, Dajian Zhang

**Affiliations:** 1 College of Agriculture, and State Key Laboratory of Crop Biology, Shangdong Agricultural University, Tai’an, 271018 Shandong China; 2 Crop Research Institute, Shandong Academy of Agricultural Sciences, Jinan, 250131 Shandong China

**Keywords:** Wild relatives, Breeding, *Glycine soja*, *Glycine max*, *Glycine*, Perennial

## Abstract

Genetic diversity is a cornerstone of crop improvement, However, cultivated soybean (*Glycine max*) has undergone several genetic bottlenecks, including domestication in China, the introduction of landraces to other areas of the world and, latterly, selective breeding, leading to low genetic diversity the poses a major obstacle to soybean improvement. By contrast, there remains a relatively high level of genetic diversity in soybean’s wild relatives, especially the perennial soybeans (*Glycine subgenus Glycine*), which could serve as potential gene pools for improving soybean cultivars. Wild soybeans are phylogenetically diversified and adapted to various habitats, harboring resistance to various biotic and abiotic stresses. Advances in genome and transcriptome sequencing enable alleles associated with desirable traits that were lost during domestication of soybean to be discovered in wild soybean. The collection and conservation of soybean wild relatives and the dissection of their genomic features will accelerate soybean breeding and facilitate sustainable agriculture and food production.

## Introduction

Climate change, coupled with an increasing human population, is expected to lead to global food shortages, especially for protein-rich foods. To feed a growing population of over 9 billion people by 2050, the current rate of agricultural productivity will need to increase twofold (Tilman et al. 2011; Ray et al. 2013). Thus, developing crops with higher yields has become an urgent task for scientists. The cultivated soybean [*Glycine max* (L.) Merr.], which is rich in both protein and oil, is one of the most important crop in the word (Li et al. 2008), providing ~ 50% of the word’s oilseed production (www.fao.org).

Soybean originated in China and is thought to have been domesticated from its wild progenitor *Glycine soja* Sieb. & Zucc. ~ 5000 years ago (Hymowitz, 1970; Li et al., 2008). Although abundant soybean germplasm has been collected, much of its genetic diversity has been lost during domestication, during which resulted in the production of numerous Asian landraces of soybean. More recently, selective breeding has been used to meet human needs, giving rise to ‘genetic bottlenecks’ (Hyten et al. 2006). A study by Gai and Zhao (2001) determined that 38 ancestral varieties represent 54.18% of the nuclear genetic material of the 651 soybean cultivars released in China between 1923 and 1995. The situation is even worse in North America, one of the world’s largest soybean producing areas, where soybean genetic diversity of soybean is extremely low because of founding events. Of the 45,000 unique Asian landraces collected worldwide, 80 account for 99% of the collective parentage of North American soybean cultivars released between 1947 and 1988 (Carter et al. 2004) and 79% of rare alleles (frequency < 0.10) present in the Asian landraces have been lost (Hyten et al. 2006) in landraces introduced to North America. Compared with its annual wild relative *G. soja* Sieb. & Zucc., cultivated soybean has lost ~ 50% of its sequence diversity (Hyten et al. 2006). Such low genetic diversity of domesticated germplasm not only hinders current soybean breeding and improvement efforts, but also makes this important crop vulnerable to emerging biotic and abiotic stressors, thus threatening long-term food security (Tanksley and McCouch 1997).

**Fig. 1:**
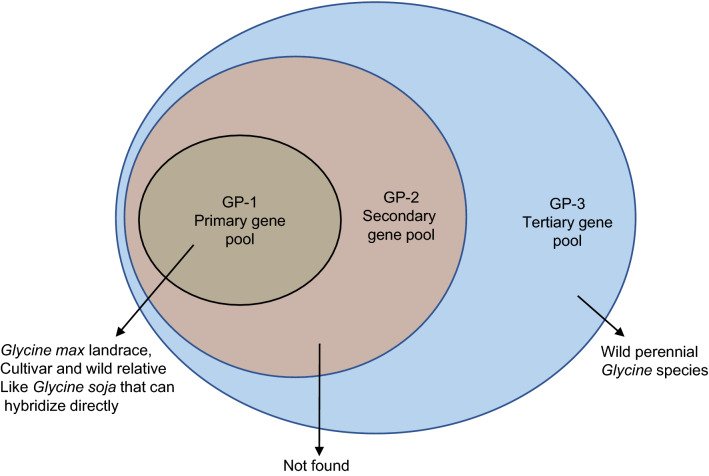
Classification of gene pools for the cultivated soybean based on Harlan and de Wet (1971)

The use of crop wild relatives of crop in traits improvements, particularly for increased pest and disease resistance, has been successful in tomato, barley, and wheat (Zhang and Batley 2020). Likewise, to overcome the bottleneck effect of cultivation in soybean, researchers have sought to broaden its gene pool using wild relatives. Harland and de Wet (1971) defined three main types of gene pools (GP), based on the success rate of hybridization among species (Fig. 1). GP-1 contains germplasm that can be easily hybridized, and includes all soybean cultivars, landraces, and its wild progenitor *G. soja* Sieb. & Zucc. GP-2 comprises species that can be crossed with GP-1 without causing F1 infertility (Dwivedi et al. 2008); however, to date, there are no soybean species in this group. GP-3 comprises 26 wild perennial species, considered to be confined to Australia. These serve as the outer limits for potential gene pools, since F1 hybridization seeds produced by crossing GP-3 species and soybean require in vitro techniques to rescue lethality (Singh et al. 2017).

Compared with cultivated soybean, wild soybean relatives have weedy, prostrate growth habits and exhibit distinct agronomic traits, such as smaller seed size, and increased pod number and seed number per plant (Hymowitz 1970; Singh et al. 2007; Singh and Hymowitz 2011). They also usually have a higher reproductive rate and increased tolerance to both biotic and abiotic stresses. Identifying the genes and alleles that are specific to wild germplasm and associated with agronomic traits is key for applying wild germplasm to broaden the genetic background and accelerate molecular breeding in soybean. Obtaining intact and accurate genomic information for wild germplasm is essential for tracking genomic variations, identifying quantitative trait loci (QTL), and conducting association studies (Xie et al. 2019).

A reference genome for the cultivated soybean accession ‘Williams 82’ was published in 2010 (Schmutz et al. 2010) and reference genomes for its annual relative *G. soja* accessions IT182932 and W05 was published by Kim et al (2011) and Xie et al. (2019), respectively. Thanks to the recent, rapid development of genome sequencing technology, and the reduction in sequencing costs, more pan-genome analyses have been conducted for both cultivated soybean plants and their wild relatives, providing abundant genome resources for soybean improvement. Here, we summarize advances in using wild soybeans as valuable resources in soybean breeding efforts, and highlight recent genomic accomplishments as well as valuable genes (Table 1) identified in both annual and perennial wild soybean species.

**Table 1:** List of genes and QTLs

Gene/locus	Function	References
*rhg1*	Soybean cyst nematode resistance	Melito et al. (2010)
*Rhg4*	Soybean cyst nematode resistance	Concibido et al. (2004)
*cqSCN-006*	Soybean cyst nematode resistance	Kim and Diers (2013)
*cqSCN-007*	Soybean cyst nematode resistance	Kim and Diers (2013)
*Rag3b/c*	Soybean aphid resistance	Zhang et al. (2013), (2017a)
*Rag6*	Soybean aphid resistance	Zhang et al. (2017b)
*Raso2*	Foxglove aphid resistance	Lee et al. (2015)
*GsWRKY20*	Drought stress	Ning et al. (2017)
*GmSALT3*	Salt stress	Guan et al. (2014)
*Ncl2*	Salt stress	Lee et al. (2009)
*GmCHX1*	Salt stress	Qi et al. (2014)
*GmTIP2*	Salt stress	Zhang et al. (2017c)
*GsGST*	Salt stress	Ji et al. (2010)
*E1*	Flowering regulation	Xia et al. (2012)
*E2*	Flowering regulation	Watanabe et al. (2011)
*E3*	Flowering regulation	Watanabe et al. (2009)
*E4*	Flowering regulation	Liu et al. (2008)
*E5*	Flowering regulation	Liu et al. (2008)
*E6*	Flowering regulation	Cober (2011)
*E7*	Flowering regulation	Cober and Voldeng (2001)
*E8*	Flowering regulation	Cober and Voldeng (2001)
*E9*	Flowering regulation	Kong et al. (2010)
*E10*	Flowering regulation	Zhai et al. (2014)
*E11*	Flowering regulation	Wang et al. (2019)
*J*	Flowering regulation	Lu et al. (2017)
*Tof5*	Flowering regulation	Dong et al (2022)
*Tof11/12*	Flowering regulation	Lu et al. (2020)
*Tof18*	Flowering regulation	Kou et al. (2022)
*Tof1111*	Flowering regulation	Lu et al. (2017)
*Tof112*	Flowering regulation	Lu et al. (2017)

## Genetic architecture of soybean’s wild relatives

The genomes of *Glycine*-grouped species were classified into seven groups based on their ability to produce fertile hybrids, and the degree to which meiotic chromosomes pair (Sherman-Broyles et al. 2014). The annual wild relative of soybean (*Glycine soja* Sieb. & Zucc.) and cultivated soybean (*G. max* (L.) Merr.) both belong to the G genome group and have the same number of chromosomes (2*n* = 40). The first sequenced genome of *G. soja* was reported by Kim et al. (2010) for *G. soja* ‘IT182932’. Illumina short reads were used to produce a genome representing 97.65% coverage of the published *G. max* ‘Williams 82’ genome sequence. Comparative analysis of the two genomes indicated ~ 0.31% sequence variation, including single nucleotide substitutions and small insertion/deletions.

To better characterize the genomic content and evolutionary history of *G. soja*, Lam et al., (2010) sequenced 31 wild and cultivated soybean accessions at ~ 5 × coverage and identified 205,614 single nucleotide polymorphisms (SNPs) for QTL mapping and association studies, as well as marker-assisted soybean breeding. Later, Li et al. (2014) sequenced and de novo-assembled seven representative accessions with an average of > 100 × Illumina reads, and constructed a pan-genome for *G. soja*. Compared with the single accession sequenced, the pan-genome was 30.2 Mbp larger and covered 94% of the annotated assembly for *G. max* ‘Williams 82’ (v1). Besides identifying core genes shared by all seven accessions, Li and colleagues reported a set of dispensable genes exhibiting a higher rate of variation, presumably as a result of adaptation to diverse environments (Li et al. 2014).

The first reference-grade genome of *G. soja* was constructed by Xie et al. (2019) for the accession *G. soja* ‘W05’. This used a combination of Illumina short reads and PacBio long reads, and enabled the identification of large structural variations and gene copy number variations. Through genome-wide comparison of the wild species ‘W05’ and the cultivated soybean accession ‘Williams 82’, they successfully identified an inversion associated with seed coat color during domestication, thus demonstrating the importance of high-quality genome assembly. In 2020, a milestone was reached on the way to unraveling the species-wide genetic diversity of annual soybeans, with the publication of the complete genome sequences for 26 representative accessions. In addition, 2898 accessions were deeply re-sequenced, including 103 annual wild soybeans (Liu et al. 2020). Comparison of annual wild relatives to cultivated soybeans identified several structural variations and candidate genes associated with domestication, such as seed coat pigmentation, thus providing valuable resources for future soybean breeding.

**Fig. 2:**
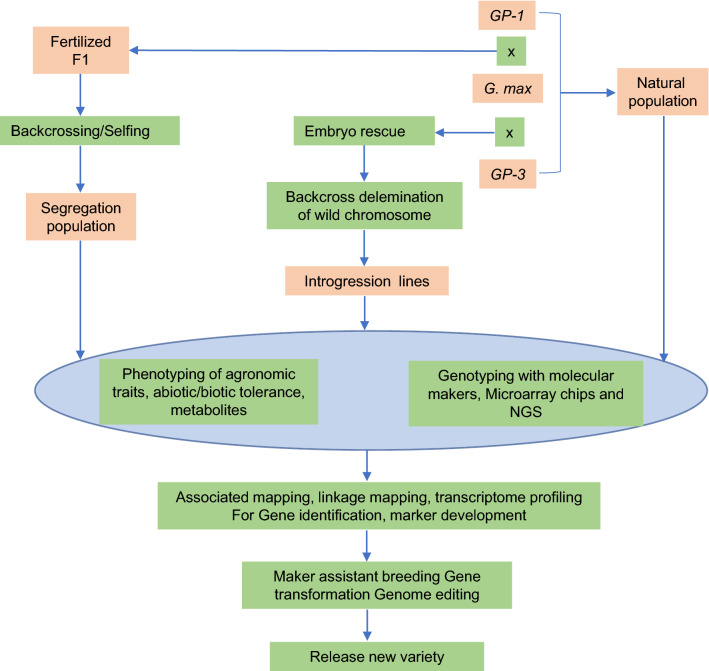
Pipeline of utilizing wild soybeans in the genetic improvement of *G. max*

The subgenus *Glycine* contains ~ 30 perennial species indigenous to Australia (Sherman-Broyles et al. 2014) that are geographically and reproductively isolated from *G. max* and *G. soja*. Compared with the annual wild relatives of soybean, the genomes of its perennial wild relatives are much more complex, belonging to six different genome groups (A–F). Unlike the subgenus *Soja*, members of the perennial subgenus *Glycine* have 2*n* = 38, 40, 78, and 80 chromosomes. Except for *G. tomentella* (2*n* = 78), hybrids of the subgenera *Glycine* and *Soja* are infertile, which making their application in conventional soybean breeding challenging. Therefore, much effort has been directed toward analyzing the annual relatives of soybean, even though many valuable agronomically related traits have been identified in the perennial species. However, this limitation could be resolved via the rapid progress of biotechnology, including improved genetic transformation and cutting-edge genome editing methods (e.g., Kim et al. 2017; Fig. 2). After the release of the genome sequences of various *G. soja* accessions, genetic analysis revealed lower heterozygosity in *G. soja* than expected, even for selfing species (Sherman-Broyles et al. 2014; Guo et al. 2012; He et al. 2012). Hyten et al. (2007) also reported that the genetic diversity of *G. soja* is approximately twofold to fivefold lower than that of the wild relatives of other plants, such as the wild brassica *Arabidopsis thaliana* (Schmid et al., 2005), wild barley (Morrell et al., 2005), and wild maize relatives (teosinte) (Wright et al., 2005), the genetic diversity of *G. soja* is about twofold to fivefold lower. Thus, the genetic diversity of cultivated soybean is not only depauperate because of domestication; but the bottleneck effect also seems to have occurred in its annual wild relatives following their speciation. In contrast, perennial wild soybeans are more phylogenetically diversified and adapted to various habitats. Therefore, identifying the genes underlying traits of interest in those species will be useful for the genetic modification of cultivated soybean.

Although efforts have been made to explore the genomic features of perennial soybeans, their genomic analysis is far behind that of their annual relatives and has been focused mainly on relatively small genomic segments because of the lack of complete reference sequences. The small segments studied to date include a 1-Mbp syntenic region surrounding the *Rpg-1*b locus on chromosome 13 (Innes et al. 2008; Wawrzynski et al. 2008), and 6–350 kb regions studied in the SoyMapII project. The first whole genome assembly of the subgenus *Glycine* was reported by Liu et al. (2018). They sequenced and assembled the *G. latifolia* genome using 10× Genomics linked-reads, representing 83.2% of the estimated genome. Synteny analysis found high genome collinearity for 12 chromosomes, while large rearrangements were identified for the other eight chromosomes. Analysis of gene families identified 3,148 *G. latifolia*-specific gene families, and genes related to defense responses, such as NB-ARC-encoding genes, were found to be overrepresented, presumably contributing to its excellent disease resistance. We recently sequenced and constructed an almost-completed reference genome for five perennial *Glycine* species, including one representative species for each genome group A–D and F, and built a pan-genome for the subgenus *Glycine*. Combining our data with the 26 pan-genomes of annual soybean constructed by Liu et al. (2020), we identified ~ 5000 core genes shared by all five perennial *Glycine* species. We also identified more than 60,000 non-core genes specific to the subgenus *Glycine*, which were either deleted or pseudolized in the subgenus *Soja* after they split from their common ancestors. An analysis of the ratio of nonsynonymous to synonymous changes (*K*_a_/*K*_s_) revealed a considerable number of genes showing signs of adaptive evolution. These genes are likely to be associated with the unique features of the subgenus *Glycine*, thus serving as potential genetic resources for soybean improvement.

## Beneficial traits of wild soybean relatives

### Biotic stress resistance

Soybean cyst nematode (SCN, caused by the pathogen *Heterodera glycines*) is the most devastating pathogen threatening soybean production, causing ~ 2.2% yield losses in Argentina, Brazil, and the America (Hartman 2015; Niblack and Riggs 2015; Hartman et al. 2017). Two major QTLs for breeding SCN-resistant soybean have been identified in *G. max*: *rhg1* on chromosome 18 (Melito et al. 2010), and *Rhg4*, located on chromosome 8 (Concibido et al. 2004) are two major QTLs for breeding SCN-resistant soybean. However, nematodes have rapidly adapted to overcome the resistance provided by these two loci. To identify novel genes conferring SCN resistance, soybean breeders have screened a large portion of *G. soja* accessions for novel SCN resistance loci. Through QTL mapping and association analysis has identified SNPs associated with SCN infection, as well as two additional QTLs, located on chromosome 15 (*cqSCN-006*) and chromosome 18 (*cqSCN-007*) (Kim et al. 2011; Zhang et al. 2016, 2017c; Kofskyet al. 2018). Cumulatively, these alleles could provide a longer duration of SCN resistance than individual alleles.

Since the perennial soybean species originated from diverse geographical areas in Australia, they may possess a broader spectrum of SNC resistance. Several studies have examined SCN resistance in perennial soybean. Riggs and Schmitt (1998) evaluated nine perennial species; all had excellent SNC resistance to three different isolates of *Heterodera glycines* (HG), with little cyst production. Bauer et al. (2007) investigated 12 perennial soybeans for HG Type 0 resistance and identified immune responses in all species except *G. curvata* and *G. pindacia*. Wen et al. (2017) further evaluated 223 plant introductions (PIs) of *G. tomentella* and 59 PIs of 12 other perennial *Glycines* for resistance to HG Type 0, HG Type 2, and HG Type 1.2.3. The authors also evaluated 36 PIs for their resistance to HG Type 1.2.3.4.5.6.7, a population that can overcome all known soybean resistance genes. Of the 223 *G. tomentella* PIs, 177 showed resistance to one HG type. Of the 36 PIs challenged with HG Type 1.2.3.4.5.6.7, 35 were resistant and 16 PIs were totally immune. Herman et al. (2020) evaluated 16 accessions of *G. argyrea*, *G. clandestine*, *G. dolichocarpoa*, and *G. tomentella* for resistance to HG. All of these accessions were classified as resistant, and none of the *G. tomentella* accessions produced any cysts. Thus, the perennial *Glycine* spp. represent valuable resources for the discovery of novel gene loci that provide broad-spectrum SCN resistance to enhance soybean breeding.

Besides SCN resistance, wild soybean species are also rich resources of genes conferring resistance to sap-sucking insects (Zhang et al. 2019). Two QTL, *Rag3b/c* and *Rag6* (Zhang et al., 2013; Zhang et al., 2017a, b), have been identified in *G. soja* (Lee et al., 2015) as contributing to soybean aphid (*Aphis glycines*) resistance, and one gene, *Raso2* contributes to resistance to foxglove aphid (*Aulacorthum solani*) resistance. Screens of perennial *Glycine* spp. also revealed remarkable resistance to the leaf rust fungus *Phakopsora pachyrhizi* (Burdon and Marshall 1981; Hartman et al. 1992; Herman et al. 2020), brown spot (Lim and Hymowitz 1987), Phytophthora root rot (Kenworthy 1989), powdery mildew (Mignucci and Chamberlain 1978), soybean rust (Hartman et al. 1992; Hymowitz 1995), Sclerotinia stem rot, and sudden death syndrome (Hartman et al. 2000). Future research should explore the molecular mechanisms underlying multiple disease resistance traits by phenotyping wild soybean species, developing associated molecular markers, and identifying the genes that contribute to the observed resistance.

### Abiotic stress tolerance

Drought reduces the productivity of soybean by up to 40% (Le et al. 2012). *G. soja* is commonly found in habitats with high water availability, and is thus usually not considered to be ideal material for developing drought-tolerant soybean varieties. Nonetheless, yield loss under drought stress was lower in transgenic *G. max* lines heterologously expressing *GsWRKY20* from *G. soja* than in the corresponding non-transgenic control plants (Ning et al. 2017). By contrast, members of the subgenus *Glycine* are well adapted to drought conditions (Song et al. 2016).

Kao and Tsai (1998) evaluated the water use efficiency (WUE) of one annual wild relative, *G. soja*, and two perennial wild relative species, *G. tomentella* and *G. tabacina*. Of these three species tested, *G. soja* showed the lowest WUE, while *G. tabacia* exhibited the highest, consistent with their geographic distributions (the selected *G. soja* accessions lived in wet habitats, while *G. tabacina* was collected from an arid area). Morphologically, *G. tabacina* also showed the least vertical midday leaflet angles and lowest photosynthetic sensitivity to water deficiency. Reference genomes are now available for all three of these species. Transcriptome profiling and comparison among these species would help us to identify drought stress responsive genes with potential use in breeding drought-tolerant soybean.

Soil salinization is a global issue directly affects arable lands and causes crop losses. Salt-affected soils currently account for 8% of the world’s total land area (www.fao.org). Soybean is classified as a moderately salt-sensitive crop (Munns and Tester 2008). Although germplasms with varying levels of salt tolerance have been reported (Phang et al. 2008), and the yields of sensitive soybean cultivars reduced dramatically under salt stress (Lee et al. 2009; Katerji et al. 2003). Although reverse genetics approaches have revealed many soybean genes with functions in the salt stress response, only one major QTL, located on chromosome 3, has been repeatedly identified in different populations. A gene named *GmSALT3* underlies this cloned QTL (Guan et al. 2014). Transcriptome analysis of different soybean varieties under salt stress suggested that the salt tolerance mechanism is genotype-specific. Peng et al. (2013) compared the physiological mechanisms of the *G. soja* accession ‘BB52’ with cultivated soybean ‘ZH13’, and established that the leaves of ‘BB52’ had higher relative water contents and water potentials than those of ‘ZH13’. Using annual wild relatives, several genes have been identified as contributing to salt tolerance, including *Ncl2* (Lee et al. 2009), *GmCHX1* (Qi et al. 2014), *GmTIP2* (Zhang et al. 2017c), and *GsGST* (Ji et al. 2010). Both diploid and polyploid perennial wild relatives, particularly *G. argyrea*, *G. clandestine*, *G. microphylla*, *G. tabacina*, *G. dolichocarp* and *G. tomentella*, have higher salt tolerance than even the most salt-tolerant cultivated soybean (Pantalone et al. 1997; Kao et al. 2006; Lenis et al. 2011). The molecular mechanism underlying the outstanding salt tolerance of perennial wild relative species remains unclear. The availability of the *G. tomentella* reference genome will facilitate the identification of genes contributing to salt tolerance in perennial wild relatives.

## Yield-related traits

Yield was the most important agronomic trait under selection during soybean domestication. In this respect, *G. max* is superior to its wild relatives. However, wild relatives of crops have been used for yield improvement in multiple species. For example, leaf angle is one of the most important agronomic traits contributing to improved growth densities and yield. Tian et al. (2019) created recombinant inbred lines between maize (*Zea mays*) and its wild ancestor species teosinte (*Zea mexicana*) and identified a gene named *UPA2* that regulates leaf angle in maize. The authors identified the teosinte version of *UPA2* and determined that it made leaves more upright when introduced into cultivated maize (Tian et al. 2019). In soybean, Akpertey et al. (2018) have reported that genetic introgression from perennial *G. tomentella* to cultivated *G. max* significantly increased seed yield. Other traits related to yield in genetically diverse accessions could also potentially be used in soybean breeding programs.

Photosynthetic efficiency is associated with crop yield, particularly when other factors such as salinity and cadmium stress are involved. Kao et al. (2003) evaluated photosynthetic gas exchange and chlorophyll a fluorescence in three wild soybeans (*G. soja*) and two perennial *Glycine* species (*G. tomentella* and *G. tabacina*) under salt stress. Different levels of sensitivity were observed among the three lines: photosynthesis was less affected by salt stress in the perennial *Glycine* spp. than in *G. soja* and was least affected in *G. tomentella*. Chen et al. (2013) and Xue et al. (2014) reported that the halophytic wild soybean ‘Dongying’ (*G. soja* Sieb. & Zucc. ‘ZYD 03262’) maintained higher photosynthetic activity under salt stress than cultivated soybean. Furthermore, Xue et al. (2014) determined that under salt stress, *G. soja* accumulated more Na^+^ in roots, but much less in leaves, than various *G. max* accessions. Thus, photosynthetic activity, stomatal conductance, and carboxylation efficiency were less affected by salt stress in *G. soja* than in the *G. max* accessions. The ability to maintain a steady leaf cell state might play a key role in the different salt stress responses of these accessions. Enhanced capacities for photosynthetic electron transport and for reversible forms of photoprotection have been reported in perennial allopolyploid *Glycine* spp. (Coate et al. 2012, 2013; Ilut et al. 2012); these advantages are presumably associated with their high ploidy levels. However, the molecular mechanisms underlying the enhanced photosynthetic ability in wild soybean remain to be elucidated.

The regulation of photoperiodic flowering is another important agronomic trait. Photoperiodic flowering is critical for regional adaptation and yield (Lin et al 2021), since early maturity is usually accompanied by low yields. Cultivated soybean and its annual wild relative *G. soja* are typical short-day plants that flower when the daylength is shorter than a certain threshold (Sedivy et al. 2017). Soybean cultivars are adapted for production in narrow bands of latitude (Kenworthy et al. 1989; Ohigashi et al. 2019). Varieties adapted to high latitude will bloom early when grown at low latitudes; such plants are normally short, with few pods. By contrast, varieties adapted to low latitude areas normally show delayed flowering, which prolongs vegetative growth for maximum yield potential; when grown at high latitudes, such plants cannot complete their lifecycles before winter (Lin et al. 2021). Combinations of forward and reverse genetic approaches have identified at least 18 major loci that participate in the photoperiod response in soybean, designated *E1* to *E11*, *J*, *Tof5*, *Tof11*, *Tof12*, *Tof18*, *Tof1111* and *Tof112* (Xia et al. 2012; Watanabe et al. 2009, 2011; Liu et al. 2008; Cober 2011; Cober and Voldeng 2001; Kong et al. 2010; Zhai et al. 2014; Wang et al. 2019; Lu et al. 2020; Lin et al. 2021; Dong et al. 2022; Kou et al. 2022). Among these, *E2*, *Tof5* and *Tof12* were associated with domestication (Wang et al. 2016; Lu et al. 2020; Kou et al. 2022) and show sequence diversity among cultivated soybean and its annual relative *G. soja*. Parallel selection was observed for *Tof5* loci in cultivated soybean and its annual wild relatives, both leading to adaptation to high latitudes (Dong et al. 2022). *Tof12* is a *PRR3* homeolog, loss of *Tof12* function had a significant role in adaptation of wild soybean during a phase of initial cultivation and domestication, analysis of the geographic distribution of *Tof12* variation suggested the gene may have permitted gradual expansion and improvement at the northern limit of early soybean cultivation (Lu et al. 2020). *Tof18* (*SOC1a*) was reported to be associated with latitude adaption as the *Tof18*^*G*^ allele facilitates adaptation to high latitudes, whereas *Tof18*^*A*^ facilitates adaptation to low latitudes; however, comparison of sequences of this loci between cultivated soybean and its annual wild relatives suggested it is not a domestication genes (Kou et al. 2022). Thus*, G. soja* has only limited utility for genetic improvement aimed at the geographic expansion of soybean. By contrast, the wild perennial *Glycine* spp. are found throughout the tropical and subtropical monsoonal belts of northern and eastern Australia, Papua New Guinea, and the Philippines. Species belonging to the *Glycine* subgenus are less sensitive to daylength, as suggested by Marshall and Broue (1981). Kenworth et al. (1989) evaluated the daylength sensitivity of 25 accessions of *G. tomentella* Hayata and one accession each of *G. arenaria* Tind. and *G. tabacina* (Labill.) Benth. under various day/night conditions. Accessions from North Queensland in the D4 and T2 isozyme groups were the least sensitive to daylength and produced both flower types under all photoperiods tested, offering a potential source of new germplasm for the improvement of soybean (Kenworth et al. 1989).

## Future prospects

Low genetic diversity poses a major challenge to soybean breeding. Wild relative resources can only be used to improve soybean if mapping populations are developed and if alleles associated with beneficial agronomic traits are identified through QTL mapping, association analysis, combined multiple omics approaches, and comparative genomic analysis. The utilization of wild soybean, particularly the perennial *Glycine* spp., requires further in-depth study of the molecular mechanisms underlying phenotypic traits to better facilitate soybean breeding. Constructing reference sequences and resequencing the genomes of soybean’s wild relatives will allow us to better characterize their genomic features. A coordinated effort is required to generate, analyze, and share data. Soybean breeders will only be able to make use of the available genetic resources if powerful computational resources and bioinformatics tools are developed and if databases for annual and perennial soybean are integrated.

## Data Availability

Data sharing not applicable to this article as no datasets were generated or analyzed during the current study.
